# Effect of Chronic Consumption of Sweeteners on Microbiota and Immunity in the Small Intestine of Young Mice

**DOI:** 10.1155/2019/9619020

**Published:** 2019-08-20

**Authors:** B. E. Martínez-Carrillo, C. A. Rosales-Gómez, N. Ramírez-Durán, A. A. Reséndiz-Albor, J. A. Escoto-Herrera, T. Mondragón-Velásquez, R. Valdés-Ramos, A. Castillo-Cardiel

**Affiliations:** ^1^Laboratorio de Investigación en Nutrición, Facultad de Medicina, Universidad Autónoma del Estado de México, Paseo Tollocan, Esquina Jesús Carranza, s/n, Colonia Moderna de la Cruz, C.P. 50180, Toluca, Mexico; ^2^Laboratorio de Microbiología Médica y Ambiental, Facultad de Medicina, Universidad Autónoma del Estado de México, Paseo Tollocan, Esquina Jesús Carranza, s/n, Colonia Moderna de la Cruz, C.P. 50180, Toluca, Mexico; ^3^Laboratorio de Inmunología de Mucosas, Escuela Superior de Medicina, Instituto Politécnico Nacional, Plan de San Luis y Díaz Mirón, C.P. 11340, Ciudad de México, Mexico; ^4^Departamento de Cirugía Experimental, Universidad Quetzalcoátl de Irapuato, Blvd. Arandas No. 975 Colonia Tabachines, C.P. 36715, Irapuato, Guanajuato, Mexico

## Abstract

The consumption of sweeteners has increased as a measure to reduce the consumption of calories and thus combat obesity and diabetes. Sweeteners are found in a large number of products, so chronic consumption has been little explored. The objective of the study was to evaluate the effect of chronic sweetener consumption on the microbiota and immunity of the small intestine in young mice. We used 72 CD1 mice of 21 days old, divided into 3 groups: (i) No treatment, (ii) Group A (6 weeks of treatment), and (iii) Group B (12 weeks of treatment). Groups A and B were divided into 4 subgroups: Control (CL), Sucrose (Suc), Splenda® (Spl), and Svetia® (Sv). The following were determined: anthropometric parameters, percentage of lymphocytes of Peyer's patches and lamina propria, IL-6, IL-17, leptin, resistin, C-peptide, and TNF-*α*. From feces, the microbiota of the small intestine was identified. The BMI was not modified; the mice preferred the consumption of Splenda® and Svetia®. The percentage of CD3^+^ lymphocytes in Peyer's patches was increased. In the lamina propria, Svetia® increased the percentage of CD3^+^ lymphocytes, but Splenda® decreases it. The Splenda® and Svetia® subgroups elevate leptin, C-peptide, IL-6, and IL-17, with reduction of resistin. The predominant genus in all groups was* Bacillus*. The chronic consumption of sweeteners increases the population of lymphocytes in the mucosa of the small intestine. Maybe, Bacillus have the ability to adapt to sweeteners regardless of the origin or nutritional contribution of the same.

## 1. Introduction

The prevalence of overweight and obesity has increased in recent years, currently considered a public health problem that affects adolescents and children [1]. This condition is alarming due to its association with chronic noncommunicable diseases such as diabetes mellitus, hypertension, cardiovascular diseases, and cancer [2]. Derived from this situation, several strategies have been created to combat them, one of which is the substitution of sugar and high fructose corn syrup with nonnutritive sweeteners [3]. Sweeteners are additives of natural or artificial origin that mimic the sweet taste of sugar without providing energy; they stimulate sweet taste receptors with minimal amounts, having a sweetening power of 200 to 600 times more than sucrose [4–6]. While sweeteners have been considered metabolically inert, recent data suggest that they may have physiological effects by altering glucose metabolism and increasing appetite stimulation [7]. It has been reported that mice exposed to saccharin show preference for foods with more sugar [8]. Not only do sweeteners increase the taste for sweet, they also intervene in the feeding behavior; for example, rodents exposed to sweeteners present higher feed intake and consequently a greater weight gain [9, 10]. The consumption of saccharin, sucralose and aspartame, causes alterations in the gut microbiota in rodent models, leading to greater food intake, excessive weight gain, and alterations in blood glucose [11–13].

Now, the small intestine (SI) is the place where processes of digestion and absorption of nutrients are carried out [14]. Anatomically, it contains Gut Associated Lymphoid Tissue (GALT) which interacts with the intestinal microbiota to promote multiple processes in different stages of life and thereby maintain the homeostasis of the host at that level [15]. The GALT has two compartments: lamina propria (effector site) and Peyer's patches (inductor site) which contain T and B lymphocytes that monitor the antigens that pass through the intestinal lumen and are responsible for secreting proinflammatory (IL-1, 2, 6, 7, 17, and TNF-*α*) and anti-inflammatory cytokines (IL-4, 10, 13, and TGB-*β*) [16]. Particularly, IL-17 is considered a sentinel and protector of the mucous barrier of the intestine, since it maintains its integrity, promotes the production of antimicrobial factors and the recruitment and generation of neutrophils as the first line of defense in mucosal sites [17]. The process of microbial colonization begins in prenatal life and continues after birth; it is modulated by factors including gestational age, diet, type of breast milk, type of delivery, hygiene, and exposure to antibiotics [18]. The environment and diet during the first days of life are crucial for the acquisition of the type of microbiota in adult life and the establishment of the host-bacteria symbiosis that influences the development of the immune and neurological systems [19]. Therefore, the content of the diet at early ages of life will determine to a large extent the type of microbiota and with it the predisposition or not to suffer from any pathology.

The composition of the intestinal microbiota varies according to the site of implantation; in the small intestine we can find* bacilli* of the* Firmicutes* families and* Actinobacteria*; in the colon mainly* Bacteroidetes*,* Lachnospiraceae*, and* Firmicutes * have been identified [20–22]. The gut microbiota plays a very important role in the immune response and intestinal homeostasis [23]; it participates actively in digestion by fermenting polysaccharides and producing monosaccharides and short chain fatty acids, increasing the deposition of triacylglycerides and the absorption of energy [24].

It is a fact that there are modifications at the level of the immune system of the mucous membranes and the microbiota of the small intestine depending on the age and type of diet consumed, which in turn generates changes in various metabolic parameters [25]. The effect of sweeteners in early life and how they affect the proliferation of the microbiota of the small intestine and thus the maturation of the mucosal immune system is still unknown. Therefore, in this study we considered to evaluate the effect of the chronic consumption of sweeteners on the microbiota and the immunity of the mucosa of the small intestine in recently weaned mice.

## 2. Materials and Methods

### 2.1. Study Design

An experimental study was carried out with 72 CD1 21-day old mice, free of pathogens, which were fed with Rodent Laboratory Chow® 5001 croquettes [3.02 Kcal / g] (RLChow® 5001) and water* ad libitum*. The mice were housed in cages, 4* per* group, under controlled conditions, temperature at 19 to 21°C, and light/dark cycles of 12 hours each. Animal care and experimental procedures were carried out in accordance with the standards of the Internal Regulation for the Use of Lab Animals and Ethical Investigation Committee of the Universidad Autónoma del Estado de México, UAEM (004/2018), as well as the guidelines of the Mexican Secretary of Health for the Production and Care of Lab Animals (NOM-062-ZOO-1999 Ministry of Agriculture, Mexico City, Mexico).

### 2.2. Study Groups

**Figure 1:**
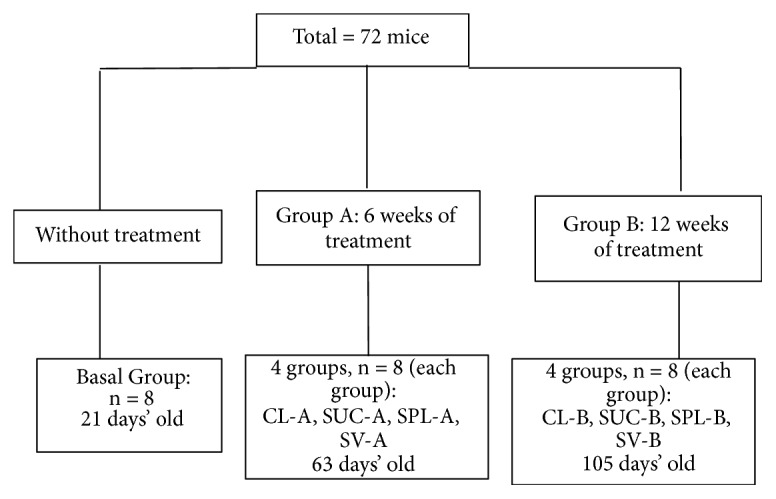
Distribution of study groups by time and type of treatment. CL (Control), SUC (Sucrose), SPL (Splenda®), and SV (Svetia®).

The 72 mice were distributed in groups (n = 8) according to the type and time of consumption of sweetener as described below (Figure 1):

- * Basal Group*: 21-day-old mice that were sacrificed at the time of weaning and never consumed RLChow® 5001 croquettes nor water supplemented with sweetener.
- * Control Group A (CL-A)*: Mice that consumed water without sweeteners for 6 weeks after weaning.
- * Sweetener groups*: Sucrose A (Sac-A), Splenda® A (Spl-A), and Svetia® A (Sv-A):
- Mice that consumed water supplemented with table sugar (Sucrose A) and commercial sweeteners Splenda® (Splenda® A) and Svetia® (Svetia® A), respectively, for 6 weeks after weaning.
- * Control group B (CL-B)*: Mice that consumed water without sweeteners for 12 weeks after weaning.
- * Sweetener groups*: Sucrose B (Sac-B), Splenda® B (Spl-B), and Svetia® B (Sv-B): mice that consumed water supplemented with table sugar (Sucrose B) and commercial sweeteners Splenda® (Splenda® B) and Svetia® (Svetia® B), respectively, for 12 weeks after weaning.

### 2.3. Administration of Sweeteners

The solution of sweeteners was prepared with ultrapure water at a concentration of 41.66 mg/mL Sucrose and 4.1 mg/mL of Splenda® or Svetia®, in accordance with the recommendations of Official Mexican Standard NOM-218-SSA1-2011 for nonalcoholic flavored drinks [26]. One envelope of Splenda® contains 1 g of carbohydrates, which includes dextrin, maltodextrin, and sucralose. One envelope of Svetia® contains sucrose, steviol glycoside (2.5g/100g), isomalt, and sucralose (0.6g/100g). The solution prepared with sweetener was placed daily in the water containers of each study group in morning from 8 to 13 h (5 hours per day); later it was removed to place water without sweetener for the rest of the day. The consumption of water with and without sweetener was quantified to determine the consumption preference.

### 2.4. Determination of the Body Mass Index (BMI)

The body mass index (BMI) was quantified weekly using the weight and length of each mouse.

- *Weight*: the mice were weighed from the beginning of the study and weekly until slaughter with a Triple Beam 700/800 Series mouse scale (Ohaus® Cat. No. 2,729,439).
- *Length*: it was determined with the mice under anaesthesia (0.1 mL of sodium pentobarbital at 1%) with a tape measure from the nose to the anus.

Once the weight and length were obtained, the BMI was calculated with the following formula [27]: BMI = Weight (g)/length (cm^2^).

### 2.5. Food and Total Energy Consumption

The calculation of food consumption was determined weekly by the difference in the amount of food at the beginning of each week less the amount of food at the end of each week of the study. The total energy intake of the mice was quantified weekly, taking into account the energy intake of each sweetener: Sucrose 4 Kcal/g, Splenda® 0 Kcal/g, and Svetia® 4 Kcal/g. The total energy consumption from food and water with sweetener* per* week was calculated with the following formula (Equation (1)) [27]:

(1) $$\begin{array}{c} Kcal=\left(\;\text{Grams of Food x 3.02}\;\right)+Sweetener\;\;Kcal \end{array}$$

### 2.6. Water Consumption with and without Sweetener

The calculation of water consumption without sweetener was done daily by the difference in the amount of water without sweetener at the beginning of the day less the amount of water without sweetener at the end of the day.

The intake of sweetener was determined daily by the difference of the amount of water with sweetener at the beginning of the exposure less the amount of water with sweetener at the end of exposure (5 hours).

### 2.7. Obtaining Samples

#### 2.7.1. Collection and Determination of Plasma Samples

The animals were euthanized by group: Basal (21 days old, without treatment); Group A, six weeks of treatment (63 days old); Group B, twelve weeks of treatment (105 days old). The animals were anesthetized with sodium pentobarbital at 1% (80 mg / kg of weight); they were bled by direct cardiac puncture (with a syringe with heparin) and sacrificed by cervical dislocation. The whole blood was centrifuged for 10 minutes at 2500 rpm to separate the two blood phases. Plasma was collected and transferred to Eppendorf tubes to quantitate leptin, resistin, C-peptide, and TNF-*α*. The determinations were made through Luminometry with a Luminex 201 from Millipore^MT^, with a commercial kit (Metabolic Magnetic Metabolic Pearl Panel, Cat. No. MMHMAG-44K) of Milliplex® Map, following the recommendations of the supplier.

#### 2.7.2. Collection of Tissue Samples and Intestinal Content

After euthanasia and obtaining total blood, the small intestine (SI) of each mouse was removed, placed in 1 mL of saline phosphate buffer 1X (PBS) to keep it hydrated until it was processed. The lumen of SI was washed with 5 mL of 1X PBS, the obtained liquid was centrifuged at 4,500 rpm for 15 minutes, supernatant was discarded, and the pellet of the intestinal contents was suspended in 1 mL of physiological solution.

### 2.8. Isolation of Lymphocytes from the Small Intestine (Peyer's Patches and Lamina Propria)

#### 2.8.1. Lamina Propria

The isolation of lymphocytes from Peyer's patches and the lamina propria of the small intestine was performed with the technique described by Reséndiz-Albor* et al.* [28] with brief modifications. Once the SI was dissected and washed, it was carefully cleaned to eliminate the mesentery and Peyer's patches were extracted. Then, an iron crochet needle of 10 cm in length was inserted with a rope tied to turn the SI. The intestine was tied at one end, the crochet needle was removed, and the rope was carefully pulled while it remained submerged in cold RPMI-1640 medium (Sigma-Aldrich, USA, Cat. R6504). Each inverted intestinal segment was transferred to a 50 mL tube, containing 25 mL of RPMI medium with 60 U/mL type IV collagenase (Sigma-Aldrich, USA, cat # C5138), DTT (1.4 Dithiothreitol, Sigma-Aldrich, USA, Cat # 43819), 1% of Fetal Calf Serum (FCS), and 50 *μ*g/ml gentamicin. The tubes were incubated horizontally for 30 minutes at 37°C in a shaking water bath at 150 rpm. The contents of each tube were then transferred to Petri dishes and 200 *μ*l of FCS was added. The intestinal mucosa was compressed with a syringe plunger on a plastic mesh. The individual suspension of cells containing lamina propria cells was filtered through an organza mesh and then centrifuged for 10 minutes at 1500 rpm at 4°C. Cell suspensions were collected and centrifuged in a discontinuous 40%/70% Percoll gradient at 2500 rpm for 25 minutes. The cells of the interface were washed and suspended in RPMI medium.

#### 2.8.2. Peyer's Patches

After being separated from the small intestine, Peyer's patches were crushed in a solution FCS/3% PBS on ice and filtered through a 300-section stainless steel cell filter to obtain lymphocytes. The cells were centrifuged for 10 minutes at 1500 rpm at 4°C.

### 2.9. Flow Cytometry Assays

The cell suspensions of Peyer's patches and lamina propria were adjusted to 1 × 10^6^ cells/mL in PBS for the cytofluorometric analysis using the technique described by Arciniega-Martínez* et al.*, with brief modifications [29]. (i) The surface phenotype of the T cells was detected using monoclonal antibodies labeled with fluorescence: anti-CD3e/PE (Cat. No. 553063), anti-CD4/PerCP (Cat. No. 553052), and anti-CD8a/APC (Cat. No. 553035) (all from BD Biosciences). The cells were incubated for 30 minutes at room temperature. Finally, the cells were washed with PBS and fixed in paraformaldehyde at 1%. (ii) For the detection of intracellular cytokine production, lymphocytes were stimulated with a mixture containing phorbol myristate acetate, ionomycin, and Brefeldin A (Leucocyte Activation Cocktail Kit, BD Pharmingen) and incubated for 4 h at 37°C and 5% CO_2_. Then, antibodies to cell surface markers, anti-CD4 PerCP (Cat.No. 553052), were added and incubated as before. For intracellular staining of CD4^+^ T cells, fixation and permeabilization were performed using Cytofix/Cytoperm Kits (BD Pharmingen) according to the manufacturer's instructions. These cells were incubated with anti-IL-6 APC (Cat. No. 561367) and anti-IL-17A FITC (Biolegend, Cat. No. 506907). The fluorescent signal intensity was recorded and analyzed by FACS Aria Flow Cytometer (Becton Dickinson). Events were collected from the lymphocyte gate on the FSC/SSC dot plot. 20,000 gated events were acquired from each sample using the CellQuest research software (Becton Dickinson). Data was analyzed using Summit software v4.3 (Dako, Colorado Inc.). Data from eight mice per group are reported as the mean ± standard deviation (SD).

### 2.10. Culture Media, Isolation, and Morphological Characterization of Bacteria

Two hundred *μ*L of the suspension obtained from the intestinal contents was inoculated into enriched culture media: HBI, Heart Brain Infusion Agar (BD Bioxon Cat. No. 255003), and BA, Blood Agar (BD BHL Cat. No. 220150). The inoculated media were incubated at 37°C for 24 to 48 hours; the most abundant and representative strains were selected and purified. The isolated strains were classified into morphological groups according to their macroscopic characteristics such as size, shape, surface, consistency, and color.

### 2.11. Identification of Species

Once the groups were formed according to the morphological characteristics, 50% of the strains of each group were selected for the genetic identification of species through a sequencing analysis of the 16S rRNA gene.

#### 2.11.1. Obtaining Biomass and DNA Extraction

To obtain biomass, the strains selected for genetic identification were inoculated by cross-stripe in HBI Agar; they were incubated at 37°C for 24 hours; the biomass was obtained by scraping and placed in 1.5 mL microtubes with 1 mL of saline solution at 0.85%. For the extraction of DNA, the Promega Wizard® Genomic commercial kit (Cat. No. A1120) was used following the manufacturer's instructions.

#### 2.11.2. Amplification of the 16S rRNA Gene

The 16S rRNA gene was amplified by Polymerase Chain Reaction (PCR); the universal primers were used:

  8f: 5′-AGAGTTTGATCMTGGCTCAG-3′
  1492r: 5′-TACGGYTACCTTGTTACGACTT-3′

The polymerase chain reaction (PCR) was carried out with the enzyme Taq polymerase (My Taq DNA Polymerase Bioline Cat. No. BIO-21105) in a Maxy Gene II Axygen Thermal Cycler (Cat. No. THERM-1001) with the following conditions: predenaturation 5 minutes at 94°C, denaturation 30 seconds at 94°C, coupling 20 seconds at 52°C, and elongation 1.5 minutes at 72°C; 34 cycles were repeated with these conditions and at the end a postelongation cycle was carried out 7 minutes at 72°C. The amplified fragments were observed on agarose gel (Pronadisa Cat. No. 8101.10) at 1% stained with ethidium bromide (BrEt, Sigma Aldrich Cat. No. E7637-1G). The amplified products of the 16S rRNA gene of selected strains were purified using the Amicon® purification kit Ultra 0.5 mL Centrifugal Filters (Millipore® Cat. No. UFC 503096) and observed in agarose gel at 1% stained with bromide ethidium to verify its quality. The purified products of the 16S rRNA gene were sent to the sequencing service of MacroGen USA Sequenciation Service, Maryland, USA.

#### 2.11.3. Sequence Analysis of the 16S rRNA Gene

The nucleotide sequences obtained were analyzed and corrected with ChromaPro Software Version 2.1.5, of Technelysium DNA Sequencing Software [30]; the consensus sequences were obtained with the software BioEdit© Biological sequence alignment editor Version 7.2.6.1 [31] and then compared with sequences deposited in the GenBank of the National Center for Biotechnology Information (NCBI) with the Basic Local Alignment Search Tool (BLAST) at https://blast.ncbi.nlm.nih.gov. [32].

### 2.12. Statistical Analysis

The data on BMI and water intake and sweetener and total energy intake were analyzed using the statistical software SPSS Version 19 for Windows; its processing was carried out through the statistical analysis of variance (ANOVA). The post hoc HSD Tukey test was performed for the comparison between groups; the differences were considered statistically significant with a value of p <0.05.

## 3. Results

### 3.1. Morphometric Parameters

#### 3.1.1. The Weight and BMI Were Not Modified with Sweetener Consumption

The mean weight of mice in the Basal group at the beginning of the sweetener supplementation was 9.27g ± 1.3. The increase in weight at 63 and 105 days old was consistent with growth and development of mice; there were no significant weight differences between the groups as shown in Table 1.

The baseline BMI was 0.195 g / cm^2^  ± 0.013; no differences were found in the study groups at 63 and 105 days old (Table 1).

### 3.2. Food Consumption and Total Energy Intake

Regarding the total energy intake and food intake, the groups of Sucrose A and Svetia® A consumed less food and therefore their total energy intake was less than the 63-day old groups; the difference was statistically significant between Control A and these study groups (Table 1). At 12 weeks of treatment, the groups of Sucrose B and Splenda® B decreased energy and food consumption, compared with the Control B group (Table 1).

### 3.3. Mice Prefer Water Consumption with Splenda® and Svetia® Than Water with Sucrose

The consumption of water without sweetener is reduced in the groups of Sucrose A and Splenda® A, compared with the Control A group (Table 2). This behavior continues until 105 days of treatment, since the groups of Sucrose B and Splenda® B consume little water without sweetener, contrary to the Svetia® B group that increases water consumption at this time.

The consumption of water with sweetener is different, since, at 6 and 12 weeks of water administration with sweetener, rodents consume a greater volume of water with Splenda® and Svetia® and reduce the consumption of water with Sucrose. It is clear that the group with Svetia® has a greater preference for water consumption with and without sweetener, but rodents consume a greater amount of Splenda®, as shown in Table 2.

### 3.4. Lymphocytes of Peyer's Patches and Lamina Propria

#### 3.4.1. After 12 Weeks of Sweetener Consumption, the Percentage of CD3^+^ Lymphocytes Was Increased in Peyer's Patches

The baseline group showed an average of 21.79%  ± 0.70 of CD3^+^ lymphocytes. This number increases after 6 weeks of treatment, particularly in the Splenda® A group (25.15%  ± 0.54), compared to the Control A and Svetia® A groups (23.1%  ± 0.118, 23.87%  ± 0.192, respectively); the Sucrose A group reduced the percentage of CD3^+^ lymphocytes (22.97%  ± 0.192). At 12 weeks of treatment the percentage of CD3^+^ lymphocytes was increased with the consumption of all sweeteners, Sucrose B (28.68%  ± 0.476), Svetia® B (28.41%  ± 1.85), and Splenda® B (27.76%  ± 0.208), compared to the Control B group (25.88%  ± 0.262).

*The Population of CD4* ^*+*^ *Lymphocytes Was Not Modified after 12 Weeks of Consumption of Splenda® and Svetia®*. The Basal group showed an average of 66.3%  ± 0.556 CD4^+^ lymphocytes. At 6 weeks of treatment, the Splenda® A group decreased the CD4^+^ population (55.74%  ± 0.535, p<0.001). The Control A group was not modified compared to the Basal Group (66.06%  ± 0.262). The groups of Sucrose A (65.94%  ± 0.861) and Svetia® A (65.77%  ± 0.406) did not show significant changes. At 12 weeks the percentage of lymphocytes from the Sucrose B group (73.03%  ± 0.123, p<0.001) decreased, compared to the Control B group (77.92%  ± 0.508), without changes in the Splenda® B groups (77.69%  ± 0.593) and Svetia® B (76.9%  ± 0.588).

*The Increase of Lymphocytes in Peyer's Patches Was Observed in CD8* ^*+*^ *Lymphocytes after Consumption of Splenda® and Svetia®*. The percentage of CD8^+^ lymphocytes of the basal group was 16.12%  ± 0.444. The Splenda® A group (16.8%  ± 0.374) increased its percentage and Svetia® A reduced it (14.72%  ± 0.572) after 6 weeks of treatment compared to Control A (15.48%  ± 0.182). At 12 weeks, the percentages of Splenda® B (12.93%  ± 0.08) and Svetia® B (12.7%  ± 0.396) increased significantly (p<0.001), compared with Control B (10.48%  ± 0.433) and Sucrose B (11.9%  ± 0.219).

#### 3.4.2. Lamina Propria

*The Svetia® Consumption Increased the Percentage of CD3* ^*+*^ *Lymphocytes, at 6 and 12 Weeks; in Contrast, the Splenda® Group after 12 Weeks of Consumption Decreased the Percentage of CD3* ^*+*^. The percentage of CD3^+^ lymphocytes in the basal group was 87.95%  ± 0.315; this value decreased significantly in the Control A group (69.8%  ± 0.588) after 6 weeks of treatment. In contrast, the groups of Sucrose A (89%  ± 0.834), Splenda® A (87.72%  ± 0.508), and Svetia® A (83.77%  ± 0.508) increased the percentage of CD3^+^ lymphocytes at 6 weeks when compared with the Control group A. At 12 weeks of consumption, the Splenda® B group showed no change in the percentage of lymphocytes (87.39%  ± 1.0) compared to the group of 6 weeks. On the other hand, when compared to the Control B groups (94%  ± 0.545), Sucrose B (95.97%  ± 0.508) and Svetia® B (94.62%  ± 0.551) were observed to be decreased, at the same time of treatment.

*The Increase of Lymphocytes in the Lamina Propria Occurs at the Expense of CD4* ^*+*^ *in the Svetia® Group*. The baseline group shows an average of 5.32%  ± 0.380 CD4^+^ lymphocytes. At 6 weeks of treatment the percentage was increased in the Svetia® A (12.83%  ± 0.187), Sucrose A (9.88%  ± 0.182), and Splenda® A (8.72%  ± 0.476) groups in that order, respectively. At 12 weeks, the increase was maintained only in the Svetia® B group (12%  ± 0.219, p<0.001); the rest of the groups remain unchanged (Control B: 9.2%  ± 0.358; Sucrose B: 9.2 %  ± 0.331; Splenda® B 9.16%  ± 0.401).

*The Percentage of CD8* ^*+*^ *Lymphocytes Was Found Depressed in the Splenda® and Svetia® Groups*. In the CD8^+^ lymphocytes of the basal group an average of 40.02%  ± 0.08 was observed. This percentage doubled in the control group A (80%  ± 0.288), after 6 weeks of treatment, but was reduced in the Splenda® A groups of (77.88%  ± 0.807) and Svetia® A (78.91%  ± 0.257) with respect to Control A. This decrease was maintained until 12 weeks of treatment in the Splenda® B groups (79.13%  ± 0.518, p<0.001) and Svetia® B (78.82%  ± 0.727, p<0.001), when compared to Control B (83.63%  ± 0.412).

### 3.5. Hormones Profile and Cytokines

*The Chronic Consumption of Splenda® and Svetia® Caused an Elevation of Leptin and C-Peptide with Reduction of Resistin. TNF-α Increased with Consumption of Svetia® but Decreased in the Splenda® and Sucrose Groups*. The baseline group showed a median of 275 pg/mL of leptin, 6700 pg/mL of resistin, 595 pg/mL of C-peptide, and 40 pg/mL of TNF-*α*. After 6 weeks of treatment, the Sucrose A and Splenda® A groups reduced the leptin concentration and increased TNF-*α*. Resistin and C-peptide were elevated (p<0.001) with the consumption of Splenda® A and Svetia® A (Table 3). At 12 weeks of treatment, leptin and C-peptide were elevated in all groups. Conversely, resistin decreased in all groups (Sucrose B, Splenda® B, and Svetia® B) and, only in the groups of Sucrose B and Splenda® B, TNF-*α* was low, as shown in Table 3.

*Cytokine Profile*

*In the Peyer Patches, IL-6 and IL-17 Were Elevated with Splenda® and Svetia® Consumption*. The percentage of cytokines IL-6 and IL-17 in the basal group was 7.9%  ± 0.016 and 1.42%  ± 0.058, respectively. At 6 weeks of treatment, IL-6 decreased its percentage in the Svetia® A, Sucrose A, and Splenda® A groups (Table 4). Conversely, IL-17 was elevated in the Splenda® A and Svetia® A groups. At 12 weeks of treatment, the percentage of IL-6 and IL-17 remained high in the Splenda® B and Svetia® B groups, compared to Control B (Table 4).

*In the Lamina Propria, IL-6 and IL-17 Were also Found Elevated with the Consumption of Splenda® and Svetia®*. The percentage of intracellular cytokines in the basal group of the lamina propria was 7.52%  ± 0.106 for IL-6 and 2.82%  ± 0.112 for IL-17. The percentage of both cytokines increased at 6 and 12 weeks of treatment with the consumption of Splenda® and Svetia®, as shown in Table 4.

### 3.6. Isolation and Morphological Characterization of Bacteria

*Isolated Strains*. 120 strains were isolated in the culture media used: 60 representative strains from HBI Agar and 60 representative strains from Blood Agar. The 120 isolated strains were distributed in 9 groups according to the macroscopic morphological differences they presented (Table 5).

### 3.7. Species Identification

The consensus sequences of the 60 selected strains were obtained to be genetically identified (50%), with a length range of 1370 to 1530 base pairs (bp). These were compared with the sequences deposited in the NCBI GenBank. It was observed that 55 of the sequences had a percentage of similarity greater than 98% and that only 5 had a percentage of similarity of 97% (Tables 6(a) and 6(b)).

We identified 14 genera and 36 different species, where the predominant genus was* Bacillus* with 14 species, followed by the genus* Pseudomonas* with 5 species and* Staphylococcus *with 4 species. In the analysis by genus and species of the strains by growth in the culture media used, it could be observed that the genera* Rothia* and* Rummeliibacillus* grew only in HBI Agar, while the genera* Acinetobacter, Arthrobacter, Kocuria, Lysinibacillus, Micrococcus, Oceanobacillus, Stenotrophomonas*, and* Streptococcus* only grew in Blood Agar. The remaining genera were obtained from both culture media (Table 7).

The bacterial growth in the different study groups was very diverse; in the Basal group it was possible to observe only the growth of four different strains:* Acinetobacter haemolyticus, Acinetobacter schindleri, Bacillus pumilus, and Rothia dentocariosa* (Table 6(a)), while, in the Control A group, the presence of* Acinetobacter haemolyticus*,* Pseudomonas koreensis*, and* Staphylococcus xylosus* was observed. However, in the Control B group, the presence of the genus* Bacillus* with the species* B. muralis, B. pumilus*, and* B. ruris* predominated, followed by* Enterococcus hirae, Lysinibacillus mangiferihumi*, and* Pseudomonas moraviensis*.

In the Sucrose A group, we observed the presence of* Lysinibacillus fusiformis*,* Pseudomonas azotoformans*, and* P. cedrina subsp fulgida*; in the genus* Staphylococcus* we observed the species* S. epidermidis*,* S. saccharolyticus*, and* S. xylosus*, and in the genus* Bacillus* predominated the species* B. aerius*,* B. safensis*,* B. subtilis*, and* B. toyonensis*. On the other hand, in the Sucrose B group were present the following:* Rummeliibacillus stabekisii*,* Micrococcus yunnanensis*,* Enterococcus hirae*, and* E. lactis*; in the genus* Bacillus* predominated again the species* B. licheniformis*,* B. megaterium*,* B. pumilus*, and* B. toyonensis*. The Splenda® A group presented a greater diversity compared to the Splenda® B group. In the Splenda® A group, the presence of* Arthrobacter albus*,* Kocuria marina*,* Micrococcus yunnanensis*, and* Pseudomonas knackmussii* was observed and the genus* Bacillus* was predominant with the species* B. asahii*,* B. atrophaeus*,* B. eiseniae*, and* B. pumilus*, while, in the Splenda® B group, only the genus Bacillus was observed with the species* B. cereus*,* B. pumilus*, and* B. safensis*. In the Svetia® A group, the presence of* Streptococcus saliviloxodontae* and the genus* Bacillus* were observed in the species* B. aerius*,* B. circulans*,* B. licheniformis*, and* B. safensis*. On the other hand, in the Svetia® B group only the presence of* Bacillus safensis*,* Oceanobacillus sojae*, and* Staphylococcus lugdunensis* was observed (Tables 6(a) and 6(b)).

The genera* Bacillus, Pseudomonas*, and* Staphylococcus* were present in most of the study groups. The genus* Bacillus* was present in 8 study groups.* B. pumilus* was observed in the Basal, Control B, Sucrose B, Splenda® A, and Splenda® B groups.* Bacillus safensis* was present in the groups: Sucrose A, Control B, Splenda® B, Svetia® A, and Svetia® B. The genus* Pseudomonas* was present in 4 groups:* P. koreensis* in the Control A group,* P. moraviensis* in the Control B group,* P. azotoformans* and* P. cedria subsp fulgida* in the Sucrose A group, and* P. knackmussii* in the Splenda® A group. The third genus with greater presence in the study groups was* Staphylococcus*; in the Control A group,* S. xylosus* was presented, in the Sucrose A group,* S. epidermidis, S. saccharolyticus*, and* S. xylosus* were presented, and in the Svetia® B group* S. lugdunensis* was presented (Table 7).

## 4. Discussion

Currently the consumption of sweeteners is increasingly popular among the population; it is believed that the consumption of artificial nonnutritive sweeteners confers certain health benefits [33]. However, other studies, such as that by Nettleton* et al.*, 2009 [34], suggest that the consumption of these sweeteners is associated with the increase in weight and the risk of suffering from type 2 diabetes mellitus (DM2). The passage of sweeteners through the gastrointestinal tract is carried out without any modification in their own structure; this situation puts them in direct contact with the intestinal microbiota, which is responsible for regulating multiple physiological functions [35].

Sucralose can be metabolized or absorbed in a minimal proportion by the intestine of mammals, but it can exert an effect on the resident bacteria of the intestine [36]. It has also been shown that sucralose can increase the secretion of serotonin, which stimulates the peristaltic and secretory activity of the intestine [37, 38].

### 4.1. The Consumption of Sweeteners Reduces the Consumption of Food and Energy

The Splenda® was consumed in greater quantity at 63 days old in the study; however, at 12 weeks of treatment the Svetia® B showed the highest consumption of sweetener. It is suggested that the intensity of sweetness increases the preference of consumption of sweet flavours and increases the appetite [39, 40]. These results agree with a study in rats, which showed that these rodents preferred to consume stevia compared to saccharin or liquid without sweetener [41], since stevia is up to 300 times sweeter than sucrose [42].

In our study, the Splenda A and B and Svetia A and B groups modified the consumption of food, thus reducing energy consumption in both cases. This is opposed to the results reported by Wang* et al.*, in 2016 [43], where they show that the consumption of sucralose increases the food intake in mice and flies mainly by two mechanisms of action, by direct stimulation to receptors with a sweet taste or, indirectly, through taste-independent neuronal mechanisms.

### 4.2. The Supplemented Groups Did Not Modify the BMI

The sweetener groups did not show changes in the BMI; these results are contrary to those reported in other studies. For example, Mattes and Popkin, in 2009 [44], concluded that the use of nonnutritive sweeteners increases BMI in healthy sedentary subjects. In contrast, in 2010, Yang Q [45] reported that healthy people who consumed beverages with three different sweeteners presented in the long term an increase in BMI with respect to those people who consumed liquid without sweetener. This can be explained by the time of exposure to sweeteners which were placed for a period of 5 h in the morning and consumed water without sweetener the rest of the time. The mice had access to water consumption with and without sweetener; it shows a predilection for the consumption of solution with sweetener, mainly Splenda and Stevia, but they consumed less amount of food, which justifies that the body weight was not modified and therefore the BMI. In addition, the CD1 mice are not an obesogenic strain, and reason for the BMI was not increased [46].

This contrasts with the study conducted by Abou-Donia* et al.*, in 2008 [11]; they reported that rodents that consumed 100 mg/kg of Splenda® weight for 12 weeks had weight gain compared to rats that did not receive Splenda® or received higher doses (300, 500, and 1000 mg/kg). This suggests that the modification of body weight does not depend exclusively on the consumption of sweeteners; rather it is a multifactorial process that depends on factors such as the type of diet, the amount and type of sweetener consumed* per* day, and the level of physical activity.

### 4.3. Sweeteners, Cytokines, Immunity, and Microbiota of the Mucosa of the Small Intestine

Studies on nonnutritive sweeteners are related to the safety of their consumption and their probable long-term effects. Most focus on their systemic effect such as risk of cancer, diabetes, dental caries, hypertension, and glycaemic control, appetite, and food [47]. In this study, we looked at the perspective of the effect of sweeteners in the modification of the percentage of lymphocytes, the secretion of cytokines and how the microbiota of the small intestine is modified as a site of contact, and absorption of sweeteners.

The findings are relevant because, after 12 weeks of sweetener consumption, the percentage of CD3^+^CD8^+^ lymphocytes was increased in Peyer's patches, after consumption of Splenda® and Svetia®. In Peyer's patches, IL-6 and IL-17 were elevated with Splenda® and Svetia® consumption. In the lamina propria, the Svetia® consumption increased the percentage of CD3^+^CD4^+^ lymphocytes, at 6 and 12 weeks. The Splenda® group after 12 weeks of consumption decreased the percentage of CD3^+^ at the expense of CD8^+^. The percentage of CD8^+^ lymphocytes was found depressed in the Splenda® and Svetia® groups. In the lamina propria, IL-6 and IL-17 were also found elevated with the consumption of Splenda® and Svetia®. This is associated with elevation of leptin and C-peptide, as well as reduction of resistin, after the chronic consumption of both Splenda® and Svetia®. On the other hand, the increased TNF-*α* with the Svetia® may cause an increase in the inflammatory state, due to an increase in TNF-*α* and C-peptide. Splenda® consumption seems to improve inflammatory status, since it decreases TNF-*α*, but it increases leptin and C-peptide.

**Figure 2:**
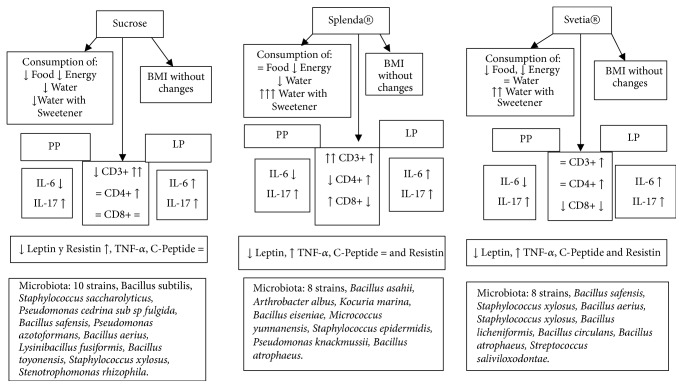
Representing the integration of quantified parameters after 6 weeks of supplementation with sweeteners. Peyer patches (PP), lamina propria (LP), interleukin-6 (IL-6), interleukin-17 (IL-17), and Cluster of Differentiation 3, 4, 8 (CD3+, CD4+, and CD8+).

**Figure 3:**
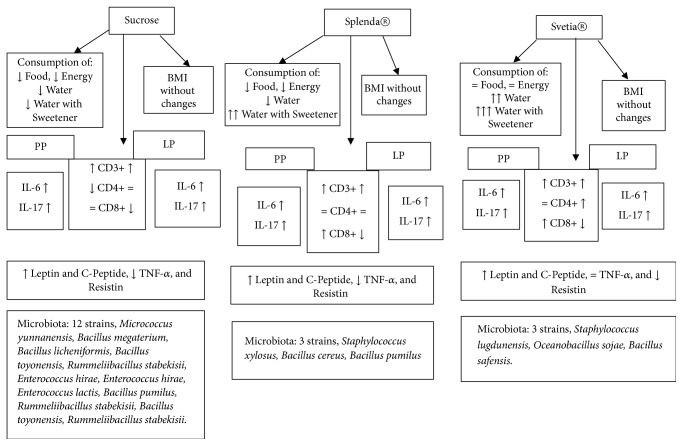
Representing the integration of quantified parameters after 12 weeks of supplementation with sweeteners. Peyer patches (PP), lamina propria (LP), interleukin-6 (IL-6), interleukin-17 (IL-17), and Cluster of Differentiation 3, 4, 8 (CD3+, CD4+, and CD8+).

While the lymphocytes of Peyer's patches and lamina propria are increased with the consumption of sweeteners, the microbiota of the small intestine is depressed substantially after 12 weeks of consumption. The gut microbiota plays a crucial role in the metabolism and immunity of the individual [48]; it cooperates with the immune system promoting signs of maturation of immune cells [34]; therefore the decrease of the microbiota can stimulate the production of lymphocytes in the mucosa of the small intestine, increasing the percentage of lymphocytes as happened in this study, as shown in Figures 2 and 3.

As is known, the composition and function of the microbiota are modulated by the type of diet consumed [35]. In this study, it can be seen how the chronic consumption of sweeteners modifies the microbiota of the small intestine.

In addition to the above, recently Uebanso* et al.,* in 2017 [49], reported that mice supplemented with sucralose for 8 weeks at doses of 1.5 and 15 mg / kg of weight did not modify their body weight, compared with mice that did not consume sucralose. They also observed that the number of bacteria from phylum* Firmicutes* and* Bacteroidetes* was similar in all mice, but* Clostridium of the XIVa* group decreased in a dose-dependent manner with the consumption of sucralose.

In this study, we can see the modification of the microbiota with chronic consumption of sweeteners; in the newly weaned Basal group, we identified 6 strains corresponding to three different genera:* Acinetobacter, Bacillus*, and* Rothia*, where the genus* Acinetobacter* predominated, while, in the Control A group, 4 strains were identified that corresponded to the genera:* Pseudomonas, Acinetobacter*, and* Staphylococcus*, where the genus* Staphylococcus* had greater predominance. In contrast, in the Control B group, 6 strains were identified, which belong to the genera:* Enterococcus, Bacillus, Lysinibacillus*, and* Pseudomonas*, showing 3 different genera with respect to the Control A group, being the* Bacillus* genus the one that predominated at 12 weeks of supplementation (Control B).

On the other hand, the groups supplemented with sucrose showed a greater diversity of identified genera. In the group Sucrose A, 10 strains belong to the genera* Bacillus*,* Staphylococcus, Pseudomonas, Lysinibacillus*, and* Stenotrophomonas*, predominantly the genus* Bacillus*, while, in the group Sucrose B, the number of identified strains was increased to 12, which belong to the genera:* Micrococcus, Bacillus, Rummeliibacillus*, and* Enterococcus*, where the genus* Bacillus* predominated followed by the genus* Rummeliibacillus*.

In the Abou-Donia* et al.* study, in 2008 [11], they collected feces from rats that consumed Splenda® for 12 weeks, cultured them on selective media for aerobic and anaerobic bacteria, and found that the rats that received Splenda® at doses of 100 mg/kg of weight had a decrease of 48.9% of anaerobic bacteria, 36.9% of* bifidobacteria*, 39.1% of* lactobacillus*, and 65.7% of* Bacteroides* compared to rats that did not consume Splenda®. In contrast, at higher doses of Splenda® (300, 500, and 1000 mg/kg of weight) they could observe a decrease of 51.2% to 67.8% in the number of aerobic bacteria with respect to rats that did not receive Splenda®.

Our results showed that, in the Splenda® A group, 8 strains were identified from genera:* Bacillus, Micrococcus, Staphylococcus*, and* Pseudomonas*, with the* Bacillus* genus being the most predominant. In the case of the Splenda® B group, the number of strains identified decreased to 3, which belong to the genera* Bacillus* and* Staphylococcus*, where the genus* Bacillus* predominated again.

It has also been reported that certain soluble extracts of the* Stevia Rebaudiana* leaf can inhibit the growth of some bacteria and fungi [50]. Other researchers mention that the metabolite of* Rebaudioside A*,* steviol*, may be responsible for the antimicrobial activity of stevia; however they do not establish any mechanism by which they exercise this activity [51, 52].

Our findings show that, in the Svetia® A group, 8 strains were identified: genera* Bacillus, Staphylococcus*, and* Streptococcus*, where* Bacillus* predominated. On the other hand, in the Svetia® B group, a decrease was observed in the number of strains identified (3 strains), which belong to two different genera:* Bacillus* and* Staphylococcus*.

In the study conducted by Li* et al.*, in 2014 [53], they evaluated the* in vitro* effect of Rebaudioside A on bacterial growth and observed that Rebaudioside A can inhibit the growth of* S. Aureus* and stimulate the growth of* L. plantarum*. These results indicate that Rebaudioside A can inhibit the growth of pathogenic microorganisms and stimulate the growth of probiotic microorganisms.

In 2015, Daly* et al.*[23] studied the composition of the microbiota of cecal content of pigs by pyrosequencing 16S rRNA gene and identified 25 families of bacteria that included 7 classes:* Bacteroidia, Clsotridia, Bacilli, Actinobacteria, Fibrobacteria, Erysipelotrichia*, and* Proteobacteria*, where* Bacteroidia, Clostridia*, and* Bacilli* predominated. The families that predominated of these three classes were* Prevotellaceae, Porphyromonadaceae, Lachnospiraceae, Ruminococcaceae, Veillonellaceae*, and* Lactobacillaceae*. When comparing these results with the microbiota of pigs supplemented with SUCRAM (neohesperidin dihydrochalcone and saccharin), they found a higher abundance of the populations of* Ruminococcaceae, Veillonellaceae*, and mainly of* Lactobacillaceae*; they concluded that the consumption of this high intensity sweetener significantly modified the composition of the intestinal microbiota.

Suez* et al.*, in 2014 [36], reported that the consumption of saccharin alters the composition of the intestinal microbiota in mice inducing intolerance to glucose. These mice showed a marked dysbiosis in comparison with mice that did not consume saccharin, with an increase of bacteria belonging to the genus* Bacteroides, Clostridiale*s, and* Lactobacillus reuteri*.

The fermentation of artificial sweeteners by the gut microbiota is not yet fully established, although the current reports are based on the fermentative capacity of the colon microbiota on sugars and other compounds in the diet [54]. Other studies report that steviol is fermented by bacteria present in the colon; this fermentation is mediated mainly by the genus Bacteroides producing steviol as the main metabolite [55, 56]. In general, most reports study the microbiota of the colon, but there are few reports that attempt to clarify its effect at the level of the small intestine and its relationship with the immune system.

Although the Bacteroides are the most abundant in the gut microbiota and the most studied at present for their ability to use glycans and form short chain fatty acids as a product of their fermentation [57, 58], it is necessary to establish the mechanisms of action by which the intestinal microbiota as a whole is able to degrade and metabolize nonnutritive sweeteners [59]. In the present study, the most abundant genus was Bacillus in all groups of the small intestine microbiota, being this the most important difference with the colon microbiota.

## 5. Conclusions

The consumption of sweeteners is not related to food intake or energy intake; the highest intake of food and energy was presented in the Control group, which had a lower water intake compared to the mice that received sweetener. The highest ingestion of sweetener is directly related to the increase in body mass index; the mice that received Svetia® ingested a greater amount of sweetener at the end of the study and presented the highest increase in BMI.

The consumption of sweeteners increases the percentage of CD3^+^CD8^+^ lymphocytes in Peyer's patches and CD3^+^CD4^+^ in the lamina propria, in addition to modifying the composition of the intestinal microbiota. The groups supplemented with sweeteners had a greater diversity of the intestinal microbiota compared with the Control groups; the sucrose A and B groups presented a greater diversity of bacteria in terms of gender and species identified.

Despite this great diversity of genera and species identified, the genus that predominated in all study groups was the genus* Bacillus*, which may suggest that the genus Bacillus may have the ability to adapt to sweeteners regardless of origin or nutritional contribution of the same.

More research is needed regarding the interaction of intestinal microbiota and sweeteners; the evidence that exists today is not clear or conclusive; this study shows the modification of bacterial diversity caused by the ingestion of one or another sweetener; however, the main metabolic pathways or mechanisms of action by which the intestinal microbiota is able to degrade the different sweeteners and use the residual metabolites are still unknown.

## Figures and Tables

**Table 1:** Morphometric values and consumption of food and energy.

	CL	SUC	Splenda®	Svetia®	
	Mean±SD	Mean±SD	Mean±SD	Mean±SD	*p*∗ Value
*Group A (63 days old)*					

Weight (g)	30.6 ± 2.82	31.8 ± 3.1	32.7 ± 2.7	32.7 ± 2.3	0.360
BMI (g/cm^2^)	0.294 ± 0.02	0.287 ± 0.013	0.298 ± 0.016	0.300 ± 0.017	0.409
Food (g)	157 ± 9.51	149.8 ± 1.17	154.3 ± 0.267	149.3 ± 5.4	*0.025∗*
Energy (Kcal)	474.4 ± 28.73	466.8 ± 2.8	466.1 ± 0.807	452.4 ± 16.3	0.086

*Group B (105 days old)*					

Weight (g)	34.4 ± 2.4	36.5 ± 2.8	37.1 ± 2.2	37.3 ± 3.046	0.148
BMI (g/cm^2^)	0.276 ± 0.013	0.289 ± 0.021	0.289 ± 0.014	0.290 ± 0.018	0.277
Food (g)	140.5 ± 11.4	125.5 ± 5.8	134.5 ± 4.2	139.4 ± 10.8	*0.007∗*
Energy (Kcal)	424.4 ± 34.7	401.6 ± 16.8	406.1 ± 12.9	424.3 ± 32.6	*0.002∗*

The values represent the Mean ± Standard Deviation (SD) of morphometric, food consumption and energy. Grams (g), centimeters (cm), grams per square centimeter (g/cm2), and Body Mass Index (BMI). One-way ANOVA was performed to compare the differences between the groups; the differences were considered statistically significant with a value of p<0.05∗.

**Table 2:** Consumption of water with and without sweetener.

	CL	SUC	Splenda®	Svetia®	
	Mean±SD	Mean±SD	Mean±SD	Mean±SD	*p*∗ value

*Group A (63 days old)*					

Water (mL)	235 ± 23.5	218 ± 6.9	221 ± 0.535	230 ± 7.7	0.053
Water with sweetener (mg/mL)	-* *-* *-	86 ± 14.1	153 ± 4.3	96 ± 5.9	*0.001∗*

*Group B (105 days old)*					

Water (mL)	227 ± 32	206 ± 7.4	218 ± 11.7	231 ± 6.4	*0.042∗*
Water with sweetener (mg/mL)	-* *-* *-	134 ± 4.4	191 ± 4	195 ± 4.4	*0.001∗*

The values represent the Mean ± Standard Deviation (SD) of water consumption with and without sweetener in milliliters. Milliliters (mL) and milligrams per milliliter (mg/mL). ANOVA was performed to compare the differences between the groups; the differences were considered statistically significant with a p value p<0.05∗.

**Table 3:** Hormone profile of CD1 mice supplemented with sweeteners for 6 and 12 weeks.

	CL	SUC	Splenda®	Svetia®	
	Median	Median	Median	Median	p∗ Value
	pg/mL	pg/mL	pg/mL	pg/mL	
*6 weeks of treatment*					

Leptin	245	181	187	456	0.069
Resistin	5026	4490	8770	8824	*0.004∗*
Peptide-C	505	508	688	1260	*0.009∗*
TNF-*α*	25	32	32	27	0.314

*12 weeks of treatment*					

Leptin	380	459	446	421	0.840
Resistin	5544	3784	4675	4788	0.149
Peptide-C	619	853	876	902	0.149
TNF-*α*	25	22	23	25	0.580

The values represent the Median concentration of hormones in pg/mL of mice supplemented with sweeteners. The nonparametric Kruskal-Wallis test was performed to compare the differences between the groups. The differences were considered statistically significant with p<0.05 ∗.

**Table 4:** Cytokine CD4^+^ T cell response from Peyer's patches and lamina propria of CD1 mice supplemented with sweeteners for 6 and 12 weeks.

	CL	SUC	Splenda®	Svetia®	
	Mean±SD	Mean±SD	Mean±SD	Mean±SD	P value∗
	(%)	(%)	(%)	(%)	
*6 weeks of treatment, Peyer's patches*					

IL-6	4.59±0.064	2.77±0.08	3.52±0.069	2.47±0.106	0.001∗
IL-17A	1.15±0.08	2.94±0.074	3.87±0.058	3.33±0.074	0.001∗

*12 weeks of treatment, Peyer's patches*					

IL-6	6.16±0.08	8.29±0.053	11±0.176	8.38±0.053	0.001∗
IL-17A	1.71±0.08	4.46±0.235	5.04±0.053	5.32±0.074	0.001∗

*6 weeks of treatment, Lamina propria*					

IL-6	9.39±0.08	10.11±0.053	16.01±0.176	11.94±0.053	0.001∗
IL-17A	3.85±0.074	7.41±0.074	7.89±0.058	11.15±0.074	0.001∗

*12 weeks of treatment, Lamina propria*					

IL-6	6.16±0.08	8.29±0.053	11±0.176	8.38±0.053	0.001∗
IL-17A	3.36±0.074	12.25±0.074	12.04±0.058	10.17±0.08	0.001∗

The values represent the mean±SD of percentage of intracellular cytokines of Peyer's patches and lamina propria in mice supplemented with sweeteners. Two-way ANOVA was performed to compare the differences between the groups. The differences were considered statistically significant with p <0.05 ∗.

**Table 5:** Macroscopic morphological characteristics of isolated strains.

Macroscopic Characteristics						
Morphological Group	Size	Shape	Surface	Consistence	Colour	Total
1	Small	Circular entire borders	Smooth	Creamy	Withe	26
2	Large	Circular rippled borders	Smooth	Dry	Beige	2
3	Medium	Circular rippled borders	Smooth	Creamy	Beige	6
4	Medium	Irregular lobed borders	Rugose	Dry	Transparent	21
5	Medium	Irregular entire borders	Smooth	Creamy	Transparent	9
6	Large	Irregular rippled borders	Rugose	Creamy	Beige	2
7	Small	Circular entire borders	Smooth	Creamy	Yellow	21
8	Large	Circular rippled borders	Flat	Creamy	Transparent	30
9	Small	Punctiform entire borders	Rugose	Creamy	Withe	3

**Table:** (a) Microbial species identified in the small intestine of CD1 mice without treatment (21 days of age) and after 6 weeks of treatment with sweeteners.

Group	Number of Identified strains	Key of strains	Fragment size (bp)	% of Similarity in BLAST	Number Access to GenBank	Identified species
Basal	6	Bsl-1	1527	99	NR_075803	*Rothia dentocariosa strain ATCC 17931*
		Bsl-2	1398	99	NR_075803	*Rothia dentocariosa strain ATCC 17931*
		Bsl-3s	1404	99	AB33945	*Bacillus pumilus strain NBRC 12092*
		Bsl-4s	1397	98	AB661448	*Acinetobacter haemolyticus strain ATCC 17906*
		Bsl-5As	1401	98	AB661448	*Acinetobacter haemolyticus strain ATCC 17906*
		Bsl-6s	1412	99	NR_025412	*Acinetobacter schindleri strain LUH 5832*

Control A	4	ClA-1	1394	99	NR_025228	*Pseudomonas koreensis strain Ps 9-14*
		ClA-1s	1399	99	AB661448	*Acinetobacter haemolyticus strain ATCC 17906*
		ClA-2	1448	98	NR_113350	*Staphylococcus xylosus strain JCM 2418*
		ClA-3s	1418	97	NR_113350	*Staphylococcus xylosus strain JCM 2418*

Sucrose A	10	SacA-2	1397	97	M87887	*Bacillus subtilis strain 168*
		SacA-2s	1401	99	NR_113405	*Staphylococcus saccharolyticus strain JCM 1768*
		SacA-3	1404	99	NR_042147	*Pseudomonas cedrina subsp fulgida strain P515/12*
		SacA-5	1385	98	AB681259	*Bacillus safensis strain NBRC 100820*
		SacA-6	1400	99	AB680322	*Pseudomonas azotoformans strain NBRC 12693*
		SacA-7s	1406	99	NR_042338	*Bacillus aerius strain 24K*
		SacA-8s	1387	99	NR_112569	*Lysinibacillus fusiformis strain NBRC 15717*
		SacA-9s	1416	98	NR_121761	*Bacillus toyonensis BCT 7112*
		SacA-10s	1410	99	NR_113350	*Staphylococcus xylosus strain JCM 2418*
		SacA-12s	1393	99	NR_028930	*Stenotrophomonas rhizophila strain e-p 10*

Splenda® A	8	SucA-1A	1399	99	NR_024817	*Bacillus asahii strain MA001*
		SucA-1s	1403	99	NR_025361	*Arthrobacter albus strain CF-43*
		SucA-2s	1404	99	NR_025723	*Kocuria marina strain KMM 3905*
		SucA-3	1399	100	NR_108906	*Bacillus eiseniae strain A 1-2*
		SucA-3s	1398	100	NR_116578	*Micrococcus yunnanensis strain YIM 65004*
		SucA-5	1376	98	FJ357586	*Staphylococcus epidermidis strain BBN 1B3-02*
		SucA-6s	1399	97	KY818992	*Pseudomonas knackmussii strain FL 80*
		SucA-7s	1415	98	NR_112723	*Bacillus atrophaeus strain NBRC 15539*

Svetia® A	8	StA-1	1408	99	AB681259	*Bacillus safensis strain NBRC 100820*
		StA -2	1409	100	NR_113350	*Staphylococcus xylosus strain JCM 2418*
		StA -1s	1409	98	NR_042338	*Bacillus aerius strain 24K*
		StA -2A	1403	99	NR_113350	*Staphylococcus xylosus strain JCM 2418*
		StA -2s	1409	98	KY174334	*Bacillus licheniformis strain DSM 13*
		StA -3	1397	98	NR_112632	*Bacillus circulans strain NBRC 13626*
		StA -3s	1404	99	NR_112723	*Bacillus atrophaeus strain NBRC 15539*
		StA -5	1407	98	NR_126178	*Streptococcus saliviloxodontae strain NUM 6306*

Total	36					

**Table:** (b) Microbial species identified in the small intestine of CD1 mice of 12 weeks of treatment with sweeteners.

Group	Number of identified strains	Key of strains	Fragment size (bp)	% of Similarity in BLAST	Number access in GenBank	Identified Strains
Control B	6	CtB-1	1401	97	NR_075022	*Enterococcus hirae strain ATCC 9790*
		CtB-2	1408	98	NR_104284	*Bacillus muralis strain LMG 20238*
		CtB-2s	1393	98	AB33945	*Bacillus pumilus strain NBRC 12092*
		CtB-3	1398	99	NR_042161	*Bacillus ruris strain R-6760*
		CtB-3s	1395	98	NR_118146	*Lysinibacillus mangiferihumi strain M-GX 18*
		CtB-4s	1398	99	NR_043314	*Pseudomonas moraviensis strain 1B4*

Sucrose B	12	SacB-2s	1400	99	NR_116578	*Micrococcus yunnanensis strain YIM 65004*
		SacB-2	1405	97	GU252112	*Bacillus megaterium strain ATCC 14581*
		SacB-3	1396	99	NR_074923	*Bacillus licheniformis strain ATCC 14580*
		SacB-3s	1395	100	NR_121761	*Bacillus toyonensis strain BCT-7112*
		SacB-4	1407	99	NR_114270	*Rummeliibacillus stabekisii strain NBRC 104870*
		SacB-4s	1396	98	NR_075022	*Enterococcus hirae strain ATCC9790*
		SacB-6s	1393	98	NR_075022	*Enterococcus hirae strain ATCC9790*
		SacB-7	1410	100	NR_117562	*Enterococcus lactis strain BT 159*
		SacB-8	1406	99	NR_118381	*Bacillus pumilus strain SBMP2*
		SacB-9	1396	99	KT719422	*Rummeliibacillus stabekisii strain MER_TA_13*
		SacB-9s	1407	99	NR_121761	*Bacillus toyonensis strain BCT-7112*
		SacB-10	1409	99	NR_114270	*Rummeliibacillus stabekisii strain NBRC 104870*

Splenda® B	3	SucB-1s	1403	99	KY992565	*Staphylococcus xylosus strain 2B*
		SucB-3s	1406	99	KY316471	*Bacillus cereus strain ZLynn1000-57*
		SucB-4	1408	99	MF040255	*Bacillus pumilus strain LMB3G14*

Svetia® B	3	StB-1s	1415	99	NR_024668	*Staphylococcus lugdunensis strain ATCC43809*
		StB -4s	1415	99	NR_112845	*Oceanobacillus sojae strain Y27*
		StB -6	1402	100	AB681259	*Bacillus safensis strain NBRC 100820*

Total	24					

**Table 7:** Distribution of genera and species identified by study groups.

Genus	Species	Basal	Control		Sucrose		Splenda®		Svetia®	
			A	B	A	B	A	B	A	B
*Acinetobacter*	*A. haemolyticus*	**+**	**+**	-	-	-	-	-	-	-
	*A. schindleri*	**+**	-	-	-	-	-	-	-	-

*Arthrobacter*	*A. albus*	-	-	-	-	-	**+**	-	-	-

*Bacillus*	*B. aerius*	-	-	-	**+**	-	-	-	**+**	-
	*B. asahii*	-	-	-	-	-	**+**	-	-	-
	*B. atrophaeus*	-	-	-	-	-	**+**	-	-	-
	*B. cereus*	-	-	-	-	-	-	**+**	-	-
	*B. circulans*	-	-	-	-	-	-	-	**+**	-
	*B. eiseniae*	-	-	-	-	-	**+**	-	-	-
	*B. licheniformis*	-	-	-	-	**+**	-	-	**+**	-
	*B. megaterium*	-	-	-	-	**+**	-	-	-	-
	*B. muralis*	-	-	**+**	-	-	-	-	-	-
	*B. pumilus*	**+**	-	**+**	-	**+**	**+**	**+**	-	-
	*B. ruris*	-	-	**+**	-	-	-	-	-	-
	*B. safensis*	-	-	**+**	**+**	-	-	**+**	**+**	**+**
	*B. subtilis*	-	-	-	**+**	-	-	-	-	-
	*B. toyonensis*	-	-	-	**+**	**+**	-	-	-	-

*Enterococcus*	*E. hirae*	-	-	**+**	-	**+**	-	-	-	-
	*E. lactis*	-	-	-	-	**+**	-	-	-	-

*Kocuria*	*K. marina*	-	-	-	-	-	**+**	-	-	-

*Lysinibacillus*	*L. fusiformis*	-	-	-	**+**	-	-	-	-	-
	*L. mangiferihumi*	-	-	**+**	-	-	-	-	-	-

*Micrococcus*	*M. yunnanensis*	-	-	-	-	**+**	**+**	-	-	-

*Oceanobacillus*	*O. sojae*	-	-	-	-	-	-	-	-	**+**

*Pseudomonas*	*P. azotoformans*	-	-	-	**+**	-	-	-	-	-
	*P. cedrina subsp fulgida*	-	-	-	**+**	-	-	-	-	-
	*P. knackmussii*	-	-	-	-	-	**+**	-	-	-
	*P. koreensis*	-	**+**	-	-	-	-	-	-	-
	*P. moraviensis*	-	-	**+**	-	-	-	-	-	-

*Rothia*	*R. dentocariosa*	**+**	-	-	-	-	-	-	-	-

*Rummeliibacillus*	*R. stabekisii*	-	-	-	-	**+**	-	-	-	-

*Staphylococcus*	*S. epidermidis*	-	-	-	**+**	-	-	-	-	-
	*S. lugdunensis*	-	-	-	-	-	-	-	-	**+**
	*S. saccharolyticus*	-	-	-	**+**	-	-	-	-	-
	*S. xylosus*	-	**+**	-	**+**	-	-	-	-	-

*Stenotrophomonas*	*S. rhizophila*	-	-	-	**+**	-	-	-	-	-

*Streptococcus*	*S. saliviloxodontae*	-	-	-	-	-	-	-	**+**	-

## Data Availability

The quantitative data used to support the findings of this study are available from the corresponding author upon request, in format of SPSS data base. The qualitative DNA data were compared and deposited in the GenBank: https://blast.ncbi.nlm.nih.gov with the access number in GenBank, located in Tables 6(a) and 6(b).
